# A Case Report and Literature Review of Mesalazine-Induced Kidney Injury in a Pediatric Patient With Ulcerative Colitis

**DOI:** 10.3389/fped.2022.808472

**Published:** 2022-05-09

**Authors:** Shuo Wang, Pengxiang Zhou, Zailing Li

**Affiliations:** ^1^Department of Pediatrics, Peking University Third Hospital, Beijing, China; ^2^Department of Pharmacy, Peking University Third Hospital, Beijing, China

**Keywords:** mesalazine, 5-aminosalicylic acid, inflammatory bowel disease, nephrotoxicity, renal injury

## Abstract

**Background:**

Mesalazine, a preparation of 5-aminosalicylic acid, is a medication widely used in clinical practice as a first-line therapy in the treatment of mild and moderate inflammatory bowel disease. However, mesalazine has nephrotoxicity and can cause adverse events in the kidney system. While these adverse reactions are very rare, they may have serious consequences.

**Case Presentation:**

The patient was a 14-year-old boy who had a 5-year history of ulcerative colitis (UC). He received mesalazine due to relapse. Abnormal urinary protein content and sterile leukocyturia were observed 2 months after the initiation of the mesalazine treatment. The urine analysis returned to normal after discontinuation of mesalazine. However, the patients' renal function worsened again after restarting mesalazine therapy. Ten cases of mesalazine-induced renal injury were identified using a systematic literature review. We found that: (1) mesalazine-induced kidney injury was more common in boys with UC; (2) all cases had proteinuria or leukocyturia; (3) kidney injury might progress to end-stage renal disease; and (4) timely withdrawal of the drug and steroid therapy might contribute to improved renal function.

**Conclusion:**

Urinalysis results and renal function should be monitored regularly in pediatric patients receiving mesalazine therapy to avoid renal insufficiency and renal failure.

## Introduction

Mesalazine, a 5-aminosalicylic acid (5-ASA) preparation, is used as a first-line therapy in the treatment of inflammatory bowel disease (IBD), especially ulcerative colitis (UC). Mesalazine has nephrotoxicity and can cause adverse events (AEs), such as interstitial nephritis (0–1%), proteinuria (0.3%), and renal failure (0–0.2%) (1). At least 10% of patients with mesalazine-induced interstitial nephritis progress to end-stage renal disease (ESRD) (2). Extraintestinal manifestations (EIMs) of IBD are present in 15% of children with UC and 20–35% of children with Crohn's disease (3). However, only a few previous studies have described renal injury in pediatric patients with IBD, including cases of glomerulonephritis, interstitial nephritis, nephrolithiasis, and renal amyloidosis (3), which are similar to those in adults. Therefore, when renal damage occurs in mesalazine-treated IBD patients, it is important to distinguish iatrogenic or pharmacogenic adverse events. In this paper, a case of pediatric ulcerative colitis with renal damage after mesalazine has been discussed and analyzed in combination with the literature to identify the causes. The results suggest that clinical pharmacists and clinicians should closely monitor urine routines and renal functions in patients with IBD treated with mesalazine.

## Clinical Data

**Figure 1:**
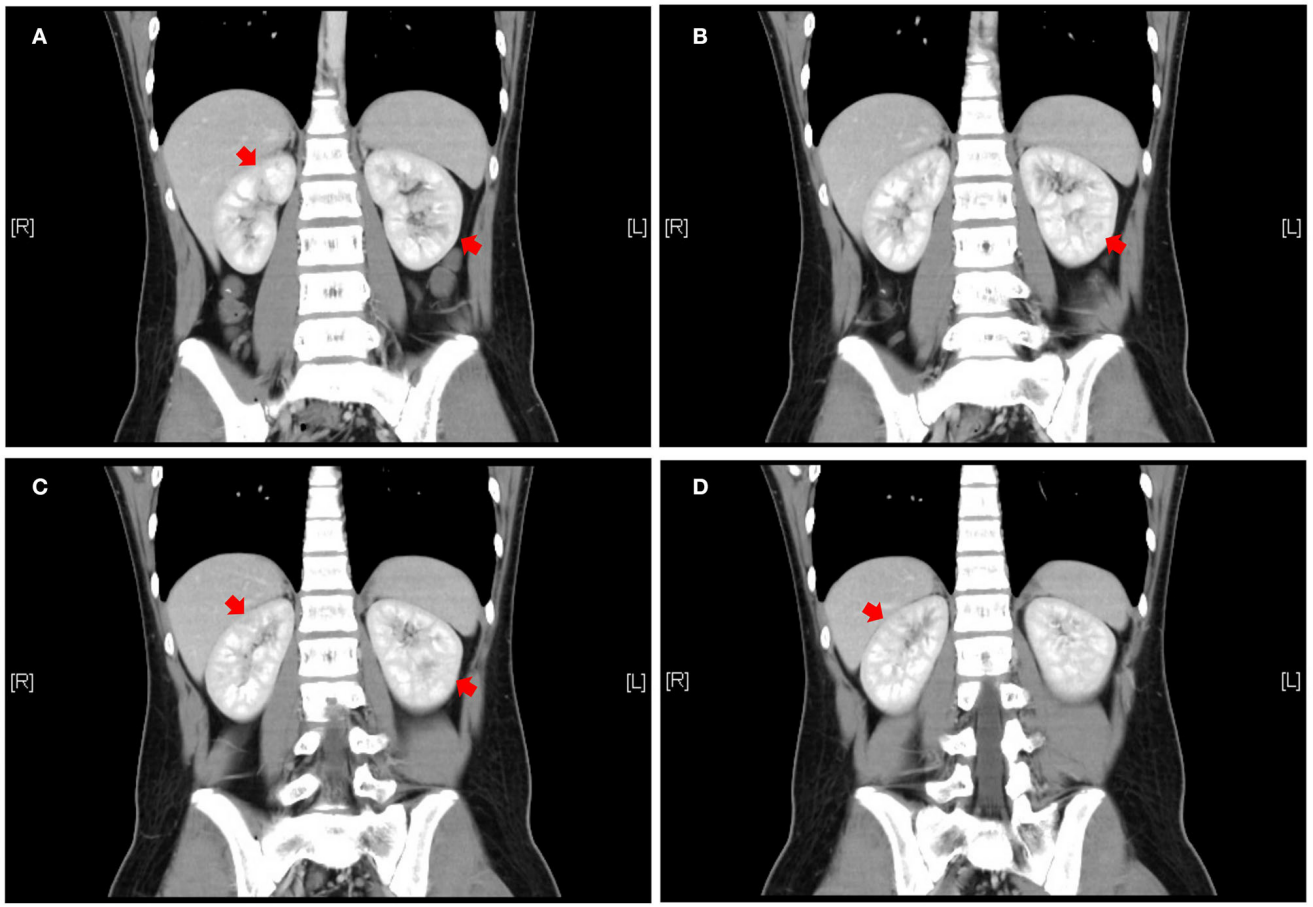
Abdominal enhanced CT coronal view, front to rear, numbered **A** to **D**. Patchy and slightly less enhanced areas are seen in both kidneys (red arrows). CT = computed tomography to illustrate the meaning of **A–D**.

The patient was a 14-year-old boy who had a 5-year history of UC. He was treated with eight doses of infliximab (5 mg/kg) and mesalazine (2 g/day) for 2 years, and he subsequently maintained clinical remission. Two years after mesalazine withdrawal, he was administered mesalazine (2.5 g/day) again owing to relapse. Leukocyturia was found after using mesalazine for 2 months and the patient denied any occurrence of fever, fatigue, nausea, hematuria, or dysuria. Physical examination revealed the following: weight, 65.3 kg; height, 172 cm; body mass index, 22.21 kg/m^2^; and blood pressure, 111/71 mmHg. No focal abdominal tenderness, rebound, or guarding were found; no edema was observed over his body. Urinalysis of the first-morning urine showed a full field of leukocytes (high power) without hematuria, bacteriuria, glucosuria, or crystalluria. Urine-specific gravity was normal. The urinary protein was 307 mg/L and protein to creatinine ratio (P/C ratio) was 0.23 mg/mg. Serum creatinine was 0.81 mg/dl and cystatin C was 0.91 mg/L. Both aerobic and anaerobic bacteria of urine cultures were negative. Serum electrolytes were normal. Kidney ultrasound did not show evidence of changes in volume, enhancement or decrease in cortical echo, or nephrolithiasis. Renal enhanced computed tomography showed uneven enhancement in both kidneys, with a locally slightly lower enhancement area (Figure 1). In addition, renal biopsy results were normal. The pediatric UC activity index (PUCAI) score was 30. Given that kidney injury may be associated with mesalazine, treatment with mesalazine was discontinued.

**Figure 2:**
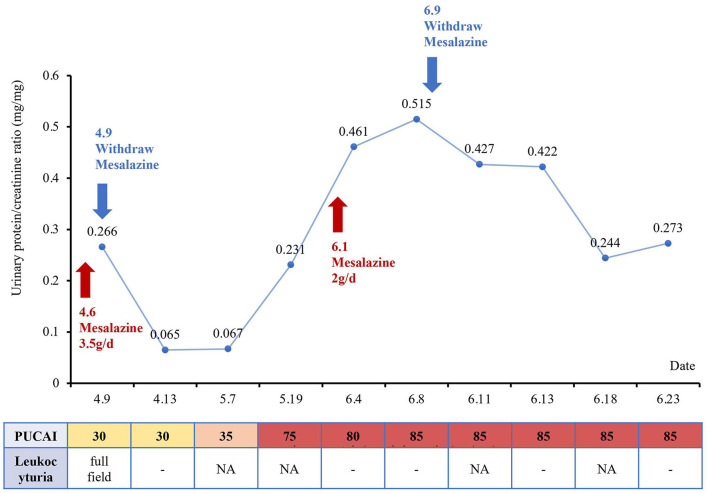
Changes in renal function and the pediatric ulcerative colitis activity index (PUCAI) score over time with the treatment of mesalazine.

Three days later, urinalysis of the first-morning urine showed no leukocyturia and urinary protein returned to normal (95 mg/L), and the P/C ratio decreased to 0.065 mg/mg. Serum creatinine was 0.38 mg/dl and cystatin C was 0.65 mg/L. However, the patient's UC symptoms began to worsen gradually. Infliximab combined with tacrolimus or cyclosporine was used to control UC, but the therapeutic efficacy was not satisfactory. Urinalysis of the first-morning urine was performed during the medication period, without leukocyturia or proteinuria. The P/C ratio fluctuated between 0.065–0.067 mg/mg, and the serum creatinine was 0.37 mg/ dL. The patient was forced to discontinue the immunotherapy due to the drug-related side effects, such as severe vomiting. The urinalysis of the first-morning urine was normal several times, and mesalazine was administered again considering the kidney injury had subsided. While kidney injury reappeared 1 week later as evidenced by proteinuria. Urinary protein was increased to 1,401 mg/L and the P/C ratio was 0.52 mg/mg. Urine culture was still negative for aerobic and anaerobic bacteria. The urinalysis of the first-morning urine returned to normal 2 weeks after mesalazine withdrawal, but the PUCAI score remained at 85 (Figure 2).

## Discussion

We use “Mesalazine,” “5-ASA,” “inflammatory bowel disease,” “kidney injury,” and “interstitial nephritis” for retrieving word search on PubMed and Cochrane Library for papers published between November 1990 and November 2021 and identified 10 pediatric cases of mesalazine-induced kidney injury (Table 1). We found that kidney injury was more common in boys (80%) with UC (70%), and mesalazine was used for 4–48 months at doses of 1.2–3 g per day at the time of diagnosis. Laboratory data showed leukocyturia in two cases and proteinuria in the other eight cases. In six cases, serum creatinine was higher than the baseline at the time of diagnosis; in nine cases, renal biopsy showed interstitial nephritis. Additionally, one case progressed to ESRD. Eight cases completely recovered after drug withdrawal and subsequent steroid treatment, and one case improved after drug withdrawal.

**Table 1:** Summary of mesalazine-induced kidney injury in pediatric patients with inflammatory bowel disease (IBD).

**Author**	**Age (years)/Gender**	**IBD type**	**Urinalysis at kidney injury diagnosis**	**Baseline creatinine (mg/dL)**	**Creatinine (mg/dL) at kidney injury diagnosis**	**5-ASA dose (g/day)**	**5-ASA treatment duration (months)**	**Steroid therapy for kidney injury**	**Outcome**
Frandsen et al. (4)	17/M	UC	WBCs, RBCs	0.7	24.6	2.4	48	No	ESRD
Uslu et al. (5)	15/F	UC	protein	0.8	1.1	1.5	4	Yes	Recovered
Arend et al. (6)	18/M	UC	WBCs	1.0	2.5	1.2	18	Yes	Recovered
Skalova et al. (7)	15/M	UC	protein	NR	1.4	3	48	Yes	Recovered
Van Biervliet et al. (8)	11/F	UC	protein	NR	0.9	1.5	36	Yes	Recovered
Alivanis et al. (9)	19/M	UC	protein	1.2	7.0	NR	7	Yes	Recovered
Benador et al. (10)	16/M	CD	protein	NR	2.5	NR	6	Yes	Recovered
	14/M	CD	protein	0.8	1.4	NR	11	Yes	Recovered
Co et al. (11)	14/M	UC	WBCs, protein	0.8	13.9	1.5	36	Yes	Recovered
Lomboy et al. (12)	13/M	CD	WBCs, protein	0.6	0.7	2	12	No	Improved
Current patient	14/M	UC	WBCs, protein	ND	0.8	2.5-3	2.5	No	Recovered

*M, male; F, female; IBD, inflammatory bowel disease; UC, ulcerative colitis; CD, Crohn's disease; 5-ASA, 5-aminosalicylic acid; ESRD, end-stage renal disease; NR, not reported; ND, no data; WBC, white blood cell*.

The pathogenesis of nephrotoxicity induced by 5-ASA remains unknown. It is generally considered to be a hypersensitivity reaction and independent of the dose and duration of treatment (2, 6). A genome-wide association study demonstrated a potential association between 5-ASA induced nephrotoxicity and HLA regions and showed that it is more common in male patients (13). The timely discontinuation of drug use and the initiation of steroid therapy can promote the recovery of renal function (9, 11). However, currently, there is a lack of randomized controlled trials of corticosteroid therapy for kidney injury.

This is the first case report of mesalazine causing kidney damage in children with UC in China. The patient with UC in this report was treated with mesalazine for 2 months. The results of urinalysis and urinary protein content were abnormal after the mesalazine treatment began, which disappeared after the discontinuation of mesalazine. It was clear that the abnormal urinalysis results were related to the mesalazine treatment. Nonetheless, there was no significant correlation between abnormal urinalysis results and aggravation of the disease. Therefore, the kidney injury was considered to be related to the mesalazine administration. Serum creatinine (from 0.37 to 0.85 mg/dL) and cystatin C (from 0.54 to 0.91 mg/L) were consistently within the normal range. The results of the renal biopsy were normal. We suggest that this patient may have benefited from the early detection of kidney injury and timely withdrawal of the mesalazine, thereby preventing the development of renal failure.

Kidney injury is a rare but severe AE of 5-ASA. It is often not diagnosed owing to its insidious onset. Therefore, it is crucial that renal function is regularly monitored in patients with IBD treated with 5-ASA. Some guidelines have included recommendations on how to monitor renal function during 5-ASA treatment for adult patients. The American Gastroenterology Society (2010) recommended that the renal function of patients should be monitored before initiating mesalazine therapy, then every 3 to 6 months for the first year, and then annually (14). The British Society of Gastroenterology consensus guidelines (2019) recommended that the renal function of patients with UC receiving 5-ASA therapy should be monitored at the baseline, repeated after 2–3 months, and then annually (15). Unfortunately, there are no recommendations for pediatric patients with IBD. Referring to the current evidence and experience from adults, children should be treated with mesalazine more cautiously, with renal function monitoring more frequently. Unfortunately, the baseline serum creatinine was not detected in this case, but the creatinine fluctuation was within the normal range during the medication period. Furthermore, the renal biopsy results were normal, which might be related to our early detection of kidney injury, preventing further kidney deterioration with timely discontinuation of mesalazine.

## Conclusion

Overall, in pediatric patients with IBD, especially in boys with UC, renal function should be assessed before 5-ASA treatment (baseline) and then monitored regularly during treatment to detect any changes. In the case of abnormal urinalysis results, an indication of renal dysfunction, or progressively higher renal function indices, 5-ASA-induced nephrotoxicity should be considered as well as EIM of IBD. Timely drug withdrawal and steroid treatment initiation may help patients to recover from kidney injury and prevent the development of renal failure.

## Data Availability Statement

The original contributions presented in the study are included in the article/supplementary material, further inquiries can be directed to the corresponding author/s.

## Ethics Statement

The study was performed according to the Declaration of Helsinki. Written informed consent was obtained from the patient's parents for publication of this case report and accompanying images.

## Author Contributions

SW: management of the patient, literature review, drafting the article, and critical revision of the article. PZ: literature review, drafting the article, data collection, and critical revision of the article. ZL: critical revision of the article. All authors read and approved the final manuscript.

## Conflict of Interest

The authors declare that the research was conducted in the absence of any commercial or financial relationships that could be construed as a potential conflict of interest.

## Publisher's Note

All claims expressed in this article are solely those of the authors and do not necessarily represent those of their affiliated organizations, or those of the publisher, the editors and the reviewers. Any product that may be evaluated in this article, or claim that may be made by its manufacturer, is not guaranteed or endorsed by the publisher.
